# Serum Mature BDNF Level Is Associated with Remission Following ECT in Treatment-Resistant Depression

**DOI:** 10.3390/brainsci12020126

**Published:** 2022-01-18

**Authors:** Marion Psomiades, Marine Mondino, Filipe Galvão, Nathalie Mandairon, Mikail Nourredine, Marie-Françoise Suaud-Chagny, Jérôme Brunelin

**Affiliations:** 1 INSERM U1028, CNRS UMR5292, Lyon Neuroscience Research Center, Université Claude Bernard Lyon 1, Université Jean Monnet, F-69500 Bron, France; marion.psomiades@ch-le-vinatier.fr (M.P.); marine.mondino@ch-le-vinatier.fr (M.M.); nathalie.mandairon@cnrs.fr (N.M.); mikail.nourredine@chu-lyon.fr (M.N.); marie-francoise.suaud-chagny@ch-le-vinatier.fr (M.-F.S.-C.); 2 CH Le Vinatier, F-69500 Bron, France; filipe.galvao@ch-le-vinatier.fr; 3 Hospices Civils de Lyon, F-69000 Lyon, France

**Keywords:** depression, BDNF, mature BDNF, ECT

## Abstract

The search for a biological marker predicting the future failure or success of electroconvulsive therapy (ECT) remains highly challenging for patients with treatment-resistant depression. Evidence suggests that Brain-Derived Neurotrophic Factor (BDNF), a protein known to be involved in brain plasticity mechanisms, can play a key role in both the clinical efficacy of ECT and the pathophysiology of depressive disorders. We hypothesized that mature BDNF (mBDNF), an isoform of BDNF involved in the neural plasticity and survival of neural networks, might be a good candidate for predicting the efficacy of ECT. Total BDNF (tBDNF) and mBDNF levels were measured in 23 patients with severe treatment-resistant depression before (baseline) they received a course of ECT. More precisely, tBDNF and mBDNF measured before ECT were compared between patients who achieved the criteria of remission after the ECT course (remitters, *n* = 7) and those who did not (non-remitters, *n* = 16). We found that at baseline, future remitters displayed significantly higher mBDNF levels than future non-remitters (*p* = 0.04). No differences were observed regarding tBDNF levels at baseline. The multiple logistic regression model controlled for age and sex revealed that having a higher baseline mBDNF level was significantly associated with future remission after ECT sessions (odd ratio = 1.38; 95% confidence interval = 1.07–2.02, *p* = 0.04). Despite the limitations of the study, current findings provide additional elements regarding the major role of BDNF and especially the mBDNF isoform in the clinical response to ECT in major depression.

## 1. Introduction

Depressive disorders are common and costly mental disorders affecting 4.4% of the world’s population according to “*Depression and Other Common Mental Disorders: Global Health Estimates*”, released by the World Health Organization (WHO, 2017) [1]. Depression is associated with severe and persistent symptoms leading to important social impairment and increased mortality. In the case of severe and/or treatment-resistant symptoms, patients can benefit from electroconvulsive therapy (ECT). In such cases, ECT shows great clinical efficacy with a remission rate of approximately 50% in patients with unipolar depressive disorder [2]. After ECT, the persistence of residual symptoms predicts a poorer long-term outcome [3]. Overall, patients who remain in a depressive episode have a poorer prognosis for their medical condition and an increased use of health services [4]. In this context, a predictive clinical or biological marker of ECT outcome would be an opportunity to improve patient care and reduce the cost of depressive disorders for the community [5]. However, the search for a biological marker predicting the future failure or success of ECT remains highly challenging [6,7].

One may hypothesize that better knowledge of the biological profile of patients who will respond may help to determine predictive markers of response. However, although the clinical efficacy of ECT is widely accepted and documented, the mechanisms by which ECT leads to a reduction in depressive symptoms remain unclear. Evidence suggests that Brain-Derived Neurotrophic Factor (BDNF), a protein known to be involved in brain plasticity mechanisms and neural survival, can play a key role in the clinical efficacy of ECT. Preclinical studies in rodents suggested that electro-convulsive shocks (ECS) may lead to an increase in Brain-Derived Neurotrophic Factor (BDNF) levels and BDNF mRNA in the brain (e.g., [8]). In addition, behavioral changes induced by ECS were positively correlated with BDNF increases [9]. In humans, the effects of ECT on BDNF levels are controversial. Although numerous studies have shown that ECT may increase BDNF levels in patients with treatment-resistant depression (TRD) [10], other studies have found no effect of ECT on BDNF levels [9]. Moreover, several studies have reported that patients who were remitters after receiving ECT had higher baseline levels of BDNF than non-remitters [11], suggesting that baseline BDNF levels may be more important in predicting remission than the ECT-induced modulation of BDNF [12]. However, the baseline differences between future remitters and non-remitters have not been observed in other studies [13,14], leaving much room for further investigation.

One of the potential confounding factors that could partly explain the controversial results reported in the literature is that previous studies only reported peripheral total BDNF levels. Indeed, the classical peripheral measure of BDNF, whether in plasma or serum, includes a combination of the three isoforms of BDNF: the BDNF precursor protein (proBDNF) and the results of its proteolytic cleavage, the mature BDNF (mBDNF) and the BDNF prodomain (truncated). Although coexisting in varying proportions, proBDNF and mBDNF elicit opposing effects on neurons. Through a high affinity with the neurotrophin receptor p75 (p75NTR), the proBDNF favours long-term depression (LTD) and apoptosis. Conversely, the mBDNF, through its high affinity with the tropomyosin-related kinase B (TrkB) receptors, favours plasticity and long-term potentiation (LTP) mechanisms [15]. These mechanisms play important roles in several physiological functions of neurons, which might be related to the pathology of mood disorders [16]. Although there is a constitutive basal secretion, the BDNF release (for review see [17]) and the respective proportion of each BDNF isoform are favoured by neuronal activation. For instance, the low-frequency stimulation of cultured hippocampal neurons preferentially induces proBDNF secretion leading to LTD, whereas high-frequency stimulation increases extracellular mBDNF leading to LTP [18]. In line with results obtained in animal models [8], it was also recently observed that the clinical effect of noninvasive brain stimulation techniques such as transcranial direct current stimulation [19] was accompanied by a modulation of mBDNF levels. Hence, thanks to its pro-plastic effects on the brain, one may hypothesize that mBDNF would be more involved than other isoforms in the beneficial long-term ECT-induced clinical effects.

The current study aimed to identify a potential predictive biomarker for the clinical efficacy of ECT treatment in patients with TRD. We hypothesized that mBDNF, given its beneficial role on neural plasticity, might be a good candidate for predicting the efficacy of ECT. We therefore investigated whether serum mBDNF levels measured at baseline could predict remission in patients with TRD receiving ECT. The baseline total BDNF level (tBDNF) corresponding to the combination of all three BDNF isoforms levels was also investigated.

## 2. Materials and Methods

### 2.1. Participants

Patients (*n* = 23) were men and women, aged from 33 years to 85 years, diagnosed with unipolar depressive disorder by the *Diagnostic and Statistical Manual of Mental Disorders* (DSM-IV-TR), and currently experiencing a major depressive episode resistant to treatment (TRD). Patients were required to have previously failed at least two adequate antidepressant trials for at least 6 weeks at therapeutic dosage and to be committed to a therapeutic procedure by electroconvulsive therapy (ECT). Patients were referred to our psychiatric unit for the treatment of patients with TRD, Le Vinatier psychiatric hospital, Bron, France between 2016 and 2019. Patients had to have been free from previous treatment with noninvasive brain stimulation, including ECT and repetitive transcranial magnetic (rTMS) or electrical (tDCS) stimulation for the current episode.

The severity of symptoms was assessed using the 10-item Montgomery–Åsberg Depression Rating scale (MADRS_10_) during psychiatric interview. Patients included met the following criteria: being older than 18 and an MADRS_10_ score > 22 at inclusion. Exclusion criteria included neurological disease, treatment with benzodiazepine, pregnancy, the presence of bipolar disorder type I or II and other comorbid Axis I diagnoses based on DSM-IV-TR criteria. All subjects provided written informed consent. This study was approved by the local ethics committee (CPP Sud EST 6, France #AU872; ANSM #2010-A01249-30). The study was preregistered in a public database on 12 January 2016 (https://clinicaltrials.gov/ (accessed on 6 December 2021) number registration: NCT02652832).

### 2.2. ECT Treatment

ECT was administered two times a week. General anaesthesia was induced with intravenous injection of either propofol (dose range = 1–1.5 mg/kg) or etomidate (0.15–0.3 mg/kg). Succinylcholine chloride (0.3–0.8 mg/kg) was used in order to prevent musculoskeletal injuries that could occur following the seizure. Bitemporal or right unilateral ECT was delivered using brief pulse stimulation (1 ms) or ultra-brief pulse stimulation (0.3 ms), respectively, using a Mecta Spectrum 5000Q (Mecta Corporation, Lake Oswego, OR, USA). ECT followed the seizure threshold titration method. ECT sessions were delivered at 6 × seizure threshold for right unilateral and 2 × seizure threshold for bitemporal placement. The length of seizure, measured by electroencephalogram, was kept over 20 s. Five patients received right unilateral ECT and 16 patients received bitemporal ECT. For 2 patients, the electrode placement was not reported in the medical record. The number of ECT sessions was determined individually on the basis of clinical observations: ECT sessions were performed until the psychiatrist considered the therapeutic response or remission was obtained or until no therapeutic benefit was observed (until a maximum of 20 sessions). During ECT course, the patients kept their pharmacological treatment unchanged and no changes in medication (dose and molecule) were allowed throughout the study period.

### 2.3. Clinical Assessments

The severity of symptoms was assessed at two time points, once before (baseline) and once after the end of ECT sessions (post-ECT) with the MADRS_10_. Remission was defined as a MADRS_10_ score < 10 [20]. The sample was divided into two groups according to their remission status after the end of the ECT course: a group of patients who achieved criteria for remission (remitters) and a group of non-remitters (MADRS_10_ > 10).

### 2.4. Biological Analyses

A 5 mL blood sample was collected from fasting patients before their first ECT session (baseline) using a serum separator tube (Vacutainer SSTTM II Advance tube). After 20 min of clotting time, the blood sample was centrifuged at 3500× *g* for 20 min to isolate the serum. The serum was then collected, aliquoted and stored at <−24 °C until assay. Participants were asked to avoid physical exercise, tobacco and alcohol consumption during the 24 h prior to the experiment in order to decrease the influence of these external factors on BDNF levels. Total BDNF and mBDNF levels were quantified by Enzyme-Linked Immunosorbent Assay (ELISA) according to the manufacturer’s instructions (BDNF Emax^®^ ImmunoAssay System, Promega Corporation, Madison, USA and mature BDNF Immunoassay, Aviscera Bioscience, Santa-Clara, USA, respectively). Briefly, serum samples were applied on precoated 96-well plates and allowed to incubate for two hours at room temperature. The reaction was stopped by stop solutions provided by the manufacturer. Plates were successively incubated with anti-human BDNF antibodies, streptavidin-HRP conjugate and substrate. The amounts of tBDNF and mBDNF were determined by measuring absorbance and calculated by comparing results with tBDNF and mBDNF curves. The absorbance was read at 450 nm with a micro-plate reader (Perkin Elmer Wallac 1420 Victor2, Winpact Scientific Inc., Saratoga, CA, USA). Intertrial reproducibility was controlled with an external standard.

### 2.5. Statistical Analyses

Comparisons between remitters and non-remitters were conducted using Fisher exact tests for categorical variables and Wilcoxon rank sum exact tests for continuous variables. Remission was a binary variable defined by an MADRS_10_ score of less than 10 after ECT sessions. Results of these comparisons were used to build the multiple logistic regression model: an alpha of 0.05 was selected as the threshold for inclusion of the variables in the regression analysis. Age and sex were added to the model as control variables. As exploratory analyses, spearman correlations were calculated to investigate the relationship between baseline BDNF levels and changes in MADRS scores. Comparisons between responders and non-responders were also undertaken. All statistical analyses were performed with R (Version 4.02).

## 3. Results

### 3.1. Sample and Clinical Effects of ECT

Demographic and clinical characteristics are summarized in Table 1.

Patients received a mean of 14.7 ± standard deviation of 4.2 ECT sessions (range 4–20). A significant therapeutic effect of the ECT course was observed in the whole sample, with a mean MADRS_10_ score reduction of 60.2% ± 20.9 (range −25.6%/100%; *p* < 0.0001). The remission rate was 30.4% (7/23 patients) and the response rate, defined as an at least 50% decrease in MADRS score from baseline, was 60.9% (14/23 patients).

### 3.2. Comparison of Remitters and Non-Remitters

As previously described, the sample was divided into two subgroups according to the remission status (MADRS_10_ < 10) after the ECT course. There was no significant difference between the two subgroups with regard to sociodemographic and clinical characteristics at baseline (see Table 2).

**Figure 1:**
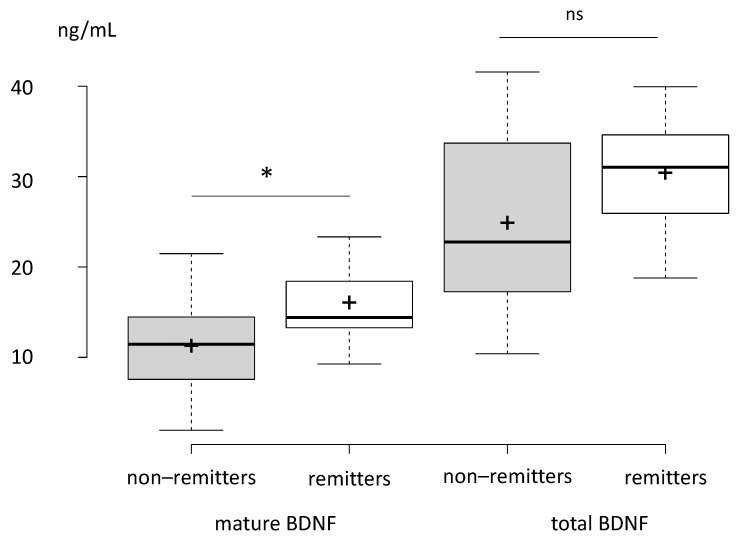
Comparison of mBDNF and tBDNF levels between patients who met the criteria for remission (remitters) or not (non-remitters) following a course of ECT. Centre lines show the medians; box limits indicate the 25th and 75th percentiles as determined by R software; whiskers extend 1.5 times the interquartile range from the 25th to 75th percentiles, outliers are represented by dots; crosses represent sample means; data points are plotted as open circles. *n* = 16, 7, 16, 7 sample points. ns: non-significant, *: *p* < 0.05.

The two subgroups significantly differed in their mBDNF levels (*p* = 0.047), but not in their total BDNF levels (*p* = 0.2), with remitters showing significantly higher baseline mBDNF levels than non-remitters (Figure 1). We therefore conducted an exploratory multiple logistic regression model analysis of association between future remission and baseline mBDNF levels (see Section 3.3).

There was no correlation between changes in MADRS_10_ scores and mature BDNF (rho = 0.035, *p* (2-tailed) = 0.874) or total BDNF (rho = 0.016, *p* (2-tailed) = 0.943). 

No differences were observed between responders and non-responders regarding total and mature BDNF levels (with response defined as an at least 50% reduction in MADRS_10_ scores from baseline). 

### 3.3. Association between Baseline mBDNF Levels and Future Remission

The exploratory multiple logistic regression model with age and sex (see Table 3) revealed that having a higher baseline mBDNF level was significantly associated with future remission after ECT sessions in patients with TRD (odds ratio (OR) = 1.38; 95% confidence interval (CI) = 1.07–2.02, *p* = 0.040).

The multivariate analysis without sex as a covariate indicated a trend toward a significant association between mBDNF and remission (OR 1.26 (95% CI 1.03–1.69; *p* = 0.053)), with no significant effect of age (OR 1.03 (95% CI 0.96 −1.12; *p* = 0.4)).

## 4. Discussion

The aim of the current study was to investigate whether baseline BDNF levels were associated with ECT outcomes in patients with unipolar TRD, and to look for an early biomarker of responses in this population. We reported that remission of depression after ECT treatment is significantly associated with a higher level of mBDNF at baseline, with no influence of both age and sex. No significant associations with tBDNF levels and remission were observed. These results suggest that high levels of mBDNF are required at baseline to obtain a clinical effect of ECT, highlighting the pivotal role of mBDNF in ECT biological mechanisms.

The current results are in line with studies reporting that BDNF is involved in the response to antidepressant treatment, and especially to antidepressant drugs [21,22,23,24,25]. As observed in the current study with mBDNF, higher levels of tBDNF pre-treatment were observed in future responders to SSRI as compared with non-responders [21]. It has also been reported that antidepressant treatments can increase BDNF levels and that BDNF level variation was correlated with clinical improvement [22]. Interestingly, retrospective studies showed that, after 2 weeks of treatment, the early non-improvement of depressive symptoms was a specific marker of final treatment failure [23] and that early changes in BDNF levels may predict the pharmacological treatment outcome [24,25]. Here, only baseline BDNF measures were investigated, and further studies are needed to investigate whether BDNF isoform proportions are modified throughout the ECT course and how early changes in BDNF isoform proportion might predict ECT clinical efficacy.

The current results are also in line with the neurotrophic hypothesis of depressive disorders postulating that depressive symptoms are associated with reduced brain plasticity [26] and that mBDNF is especially involved as compared with other isoforms [27]. BDNF is a member of the neurotrophin family of growth factors produced by neurons essential for neurogenesis during development by promoting the survival and differentiation of neurons [28], especially through its mature isoform through TrkB receptor signalling pathways. mBDNF is essential for effective synaptic plasticity in adulthood: it participates in adult neurogenesis regulation mechanisms, LTP mechanisms and promotes axonal and dendritic arborization growth (for review see [29]). These mechanisms allow for neuronal connection modulation within existing networks and facilitate the transmission of information. The present results highlighting the role of mBDNF in the response to ECT indirectly corroborate the major role of TrkB receptors in both depression and antidepressant therapies in patients with difficult-to-treat depression. Indeed, in animal models, it has been reported that the mBDNF/TrkB signalling pathway is activated following repeated sessions of ECS, while pro BDNF is not altered [30]. Moreover, TrkB-dependent neuronal differentiation has been reported to play a key role in the long-term antidepressant effects of novel antidepressant therapy such as ketamine [31]. The complex interaction between ECT and ketamine (e.g., [32]) needs further investigation to decipher the role of BDNF signalling pathways in antidepressant therapies and their combination for patients with difficult-to-treat depression. Moreover, BDNF modulates the activity of various neurotransmitters involved in the pathophysiology of depressive disorders such as glutamate, GABA, serotonin and dopamine. Current results highlight that BDNF, and particularly its mature form, is essential to allow the ECT biological effect on brain plasticity leading to clinical outcome. However, the relationship between the biological effects of ECT on neural activation [18] and BDNF isoform secretion needs further investigations.

Strikingly, the remission rate observed in the current study (*n* = 7, 30%) was below the expected values reported in the literature (e.g., between approximately 50% [2] and more than 80%, [33] depending on studies). However, it is consistent with remission rates observed in populations of patients with more severe depression, as it is in our sample [34]. Some other limitations should be acknowledged. First, we have no measurement of peripheral proBDNF levels; only total BDNF and mature BDNF were analysed in the current study, whereas it has been reported that proBDNF levels may have an influence, for instance, on the clinical effect of SSRI [35]. We also have not investigated the influence of BDNF-Val66Met-polymorphism status of participants on the current results. However, several studies have reported that BDNF-Val66Met polymorphism did not influence the clinical effects of ECT [14,36]. At a statistical level, the size of the sample and the small number of remitters after the ECT course made the estimate of the standard deviations of the coefficient associated with sex unstable in the logistic regression model. Therefore, the current results should be taken as exploratory and require a larger cohort reducing the sampling fluctuations impacting the model to be confirmed, especially because significance was not reached when sex was not entered as a covariate in the regression model. Moreover, in the current study, we measured peripheral BDNF that may not directly reflect fluctuations of BDNF in the brain. However, BDNF crosses the blood brain barrier and peripheral levels are correlated with the central rate [37]. In addition, peripheral BDNF levels correlated with depression severity: the lower the BDNF level, the greater the severity [38]. Peripheral BDNF levels are decreased in patients with depression compared with non-depressed participants as well as BDNF mRNA levels in distinct cortical areas [39,40,41].

The impacts of concurrent medication during the ECT course and illness duration are of major importance. Despite that no differences were observed in the current study between remitters and non-remitters when medication was expressed in the percent of patients taking some medication class or not, one can wonder whether medication load in terms of dose, molecule and duration may impact both ECT clinical effects and BDNF levels. Although the size of the sample and the pilot nature of the current study did not allow us to investigate these points, we encourage further research to address these questions and establish a more accurate prediction of response to ECT based on BDNF levels, medication load (in terms of class of molecule (e.g., [42]), dose and duration) and individual characteristics (such as anatomical features e.g., [43]). In the current study, five patients received right unilateral ECT (that has been associated with fewer cognitive side effects but lower clinical effect, e.g., [44]) and 16 received bitemporal stimulation. The effect of the electrodes’ placement was not investigated in the current study but requires further investigation. However, a meta-analysis reported a significant association between electrode placement and ECT-induced BDNF changes [10].

## 5. Conclusions

Despite the limitations of the study in terms of sample size and lack of pro BDNF level investigation, the current findings provide additional elements regarding the major role of mBDNF in the clinical response to ECT in patients with TRD. One may hypothesize that higher mBDNF levels are required for patients to achieve remission. Activities that allow BDNF levels in the brain to increase before entering an ECT course should be encouraged.

## Figures and Tables

**Table 1:** Demographic and clinical characteristics of the sample of patients with severe treatment-resistant major depressive disorder who received ECT.

Demographic and Clinical Characteristics	
*n*	23
Age (years)	58.0 ± 14.6
Sex (male/female)	11/12
Education (years)	12.7 ± 4.6
MADRS_10_ score at baseline	37.6 ± 5.8
Illness duration (months)	212.7 ± 197.8
Current episode duration (months)	16.9 ± 12.5
Number of previous hospitalizations	2.3 ± 1.5

Results are given as mean ± standard deviation.

**Table 2:** Characteristics of remitters and non-remitters before they received ECT.

	Non-Remitters	Remitters	*p* Value
*n*	16	7	
Female	9 (56.2%)	3 (42.9%)	0.7
Age	57.6 (16.7)	59.1 (9.4)	0.9
Education	13.5 (12.0, 16.5)	11.0 (9.0, 13.0)	0.2
Length of chronic depression (months)	114.0 (17.5, 330.0)	180.0 (144.0, 420.0)	0.2
Length of the actual episode (months)	12.0 (6.0, 18.5)	24.0 (7.5, 30.0)	0.4
Number of past hospitalizations	2.0 (1.0, 3.0)	2.0 (1.5, 3.0)	>0.9
MADRS_10_ (baseline)	39.0 (35.8, 42.2)	34.0 (31.5, 37.5)	0.13
Number of ECT	15.5 (14.0, 19.2)	12.0 (11.5, 14.5)	0.08
Delta MADRS_10_ post ECT/baseline	−19.0 (−22.2, −13.8)	−29.0 (−35.5, −23.0)	**0.005**
Baseline total BDNF (ng/mL)	22.78 (18.62, 31.73)	31.04 (25.94, 34.62)	0.2
Baseline mature BDNF (ng/mL)	11.45 (8.28, 14.26)	14.41 (13.28, 18.41)	**0.047**
Associated medication			
First generation antipsychotic	26%	21.70%	ns
atypical antipsychotic	26%	0%	ns
Other antipsychotic	8.70%	8.70%	ns
SNRI	17.40%	13%	ns
SSRI	17.40%	0%	ns
Hydroxyzine	13%	8.70%	ns
Tricyclics	17.40%	0%	ns

Results are given as median (IQR); mean (SD); *n* (%); *p*-value: Wilcoxon rank sum exact test; Wilcoxon rank sum test; Fisher’s exact test; SNRI: norepinephrine reuptake inhibitor; SSRI: selective serotonin reuptake inhibitor.

**Table 3:** Results of the multiple logistic regression model investigating the relationship between mBDNF at baseline and remission after ECT. ^1^ OR = odds ratio, CI = confidence interval.

Characteristic	OR^1^	95% CI ^1^	*p*-Value
mBDNF baseline	1.38	1.07–2.02	0.04
Age	1.01	0.92–1.11	0.8
Sex Female	—	—	
Male	6.29	0.51–162	0.2

## Data Availability

Data are available from the corresponding author on reasonable request.
